# Evaluation of Giant African Pouched Rats for Detection of Pulmonary Tuberculosis in Patients from a High-Endemic Setting

**DOI:** 10.1371/journal.pone.0135877

**Published:** 2015-10-07

**Authors:** Klaus Reither, Levan Jugheli, Tracy R. Glass, Mohamed Sasamalo, Francis A. Mhimbira, Bart J. Weetjens, Christophe Cox, Timothy L. Edwards, Christiaan Mulder, Negussie W. Beyene, Amanda Mahoney

**Affiliations:** 1 Swiss Tropical and Public Health Institute, Basel, Switzerland; 2 University of Basel, Basel, Switzerland; 3 Ifakara Health Institute, Bagamoyo, Tanzania; 4 Anti-Persoonsmijnen Ontmijnende Product Ontwikkeling (APOPO), Morogoro, Tanzania; 5 Department of Psychology, Western Michigan University, Kalamazoo, Michigan, United States of America; 6 Amsterdam Institute for Global Health and Development, Amsterdam, The Netherlands; University of Cape Town, SOUTH AFRICA

## Abstract

**Background:**

This study established evidence about the diagnostic performance of trained giant African pouched rats for detecting *Mycobacterium tuberculosis* in sputum of well-characterised patients with presumptive tuberculosis (TB) in a high-burden setting.

**Methods:**

The TB detection rats were evaluated using sputum samples of patients with presumptive TB enrolled in two prospective cohort studies in Bagamoyo, Tanzania. The patients were characterised by sputum smear microscopy and culture, including subsequent antigen or molecular confirmation of *Mycobacterium tuberculosis*, and by clinical data at enrolment and for at least 5-months of follow-up to determine the reference standard. Seven trained giant African pouched rats were used for the detection of TB in the sputum samples after shipment to the APOPO project in Morogoro, Tanzania.

**Results:**

Of 469 eligible patients, 109 (23.2%) were culture-positive for *Mycobacterium tuberculosis* and 128 (27.3%) were non-TB controls with sustained recovery after 5 months without anti-TB treatment. The HIV prevalence was 46%. The area under the receiver operating characteristic curve of the seven rats for the detection of culture-positive pulmonary tuberculosis was 0.72 (95% CI 0.66–0.78). An optimal threshold could be defined at ≥2 indications by rats in either sample with a corresponding sensitivity of 56.9% (95% CI 47.0–66.3), specificity of 80.5% (95% CI 72.5–86.9), positive and negative predictive value of 71.3% (95% CI 60.6–80.5) and 68.7% (95% CI 60.6–76.0), and an accuracy for TB diagnosis of 69.6%. The diagnostic performance was negatively influenced by low burden of bacilli, and independent of the HIV status.

**Conclusion:**

Giant African pouched rats have potential for detection of tuberculosis in sputum samples. However, the diagnostic performance characteristics of TB detection rats do not currently meet the requirements for high-priority, rapid sputum-based TB diagnostics as defined by the World Health Organization.

## Introduction

Tuberculosis (TB) is the world’s second deadliest infectious disease, which killed 1.5 million people in 2013—approximately one person every 25 seconds [1]. A key priority for TB control is the accurate and early diagnosis in persons with active and potentially infectious TB to enable timely treatment that both cures patients and decreases transmission risk. The development of novel, accurate, robust, and rapid diagnostic capabilities will result in improved case detection, disease surveillance, healthcare delivery, and quality of future research.

The superior olfactory characteristics of animals have been formerly used for diagnosis of a variety of diseases. Trained dogs are capable of identifying pulmonary carcinoma in breath samples [2] or intestinal infections in stool samples [3]. Rats have a highly developed sense of smell. The number of functional olfactory receptor genes is about 3 times larger in rats than in humans [4]. Previous investigations suggest that trained giant African pouched rats (*Cricetomys gambianus*) are able to detect and indicate the presence of *Mycobacterium tuberculosis (M*.*tb)* in sputum samples by smelling volatile organic compounds [5][6][7][8][9][10][11]. The rats presumably detect a combination of volatile organic compounds specific to M. tuberculosis, rather than a single molecule [12]. Consequently, TB detection rats have the potential to become an alternative or a supplement to sputum smear microscopy which is characterised by low sensitivity although being the only widely used TB diagnostic in resource-limited settings [13].

Previous studies on TB detection rats provided proof of concept for the test [5] and showed its value as a tool for rescreening of samples from microscopy centres (second-line screening) by increasing TB case detection after microscopy by 31.4%, 44%, and 42.8% in 2008, 2009, and 2010, respectively [6][7][8]. In those studies the TB detection rats’ performance was assessed against smear microscopy as the reference standard. A recent study has compared the accuracy of 10 rats in 910 sputum samples with the correspondent outcome from culture on solid media and subsequent multiplex polymerase chain reaction for species differentiation. The per-patient analysis showed that the mean sensitivity of the 10 rats used in this experiment was 70.5%, while the mean specificity was 80.5% [10].

However, the diagnostic potential of TB detection rats in respiratory specimen has been so far only evaluated in sputum previously evaluated in Direct Observation Treatment Short-Course (DOTS) centres accompanied by no or scarce clinical data, no follow up information, and without blinding of the involved investigators. For that reason, we have conducted the first prospective evaluation study on giant African pouched rats to detect TB in adult patients with symptoms of pulmonary tuberculosis which includes both comprehensive clinical and microbiological data. Rigorous evidence-based evaluation of diagnostic tests is essential prior to any clinical practice to avoid unwanted clinical consequences due to misleading results of test accuracy and to limit healthcare costs by preventing unnecessary testing or avoidable follow-up investigations [14][15].

## Methods

### Study population

Individuals with signs and symptoms suggestive of pulmonary TB were prospectively recruited in two cohort studies (TB Cohort and TB CHILD), and followed up for at least 5 months. The recruitment took place at the Ifakara Health Institute, Bagamoyo, United Republic of Tanzania, between the 22^nd^ of September 2010 and 8^th^ of March 2012. Bagamoyo, a coastal town of 35,000 inhabitants, is located approximately 70 km north of Tanzania’s largest city Dar es Salaam. Tanzania is one of the 22 high-burden countries with 295 prevalent bacteriological confirmed pulmonary TB cases per 100,000 [16] and 37% HIV infection in patients with TB [1].

Patients were eligible for the study if they presented with persistent cough of two weeks or more and at least one of the following TB associated findings: haemoptysis, chest pain, fever, night sweats, constant fatigue, recent unexplained weight loss, loss of appetite, malaise, or contact with a known TB case. Patients who received anti-TB treatment during the past year, were severely sick from TB or another disease, or did not reside within the study area were excluded from the study.

A minimum necessary sample size of 403 presumptive TB patients was calculated for the diagnostic study [17], assuming a prevalence of disease of 20%, an expected sensitivity and specificity of 70% and 80%, respectively, and a target accuracy of ±5% (i.e. confidence interval width of 10%) plus a type I error probability of less than 5%.

### Classification of patients

The participants were categorised into six groups based on clinical and microbiological assessments, as shown in Table 1. The allocation to the groups was not mutually exclusive, e.g. due to mixed infection of *M*.*tb* and non-tuberculous mycobacteria (NTM). For the purpose of this analysis, the classification into group A, B, D, or G supersedes classification to group C.

**Table 1:** Patient classification.

Group	Description	Short name
A	Culture-positive, smear-positive, *M*. *tuberculosis;*	s+/c+ *M*.*tb*
B	Culture-positive, smear-negative, *M*. *tuberculosis*	s-/c+ *M*.*tb*
C	Culture-positive, non-tuberculous mycobacteria	s±/c+ NTM
D	Culture-negative pulmonary TB; strong clinical and radiological suspicion	s-/c- clin.TB
F	Smear-/culture-negative, sustained recovery up to 5th month	Controls
G	Loss to follow-up after recruitment or any other combination	Indeterminate

### Study procedures and laboratory methods

Clinical procedures at enrolment comprised medical history, physical examination, voluntary HIV counselling and testing, and chest radiography. Chest radiographs were interpreted by a trained radiographer for immediate patient management.

Two sputum samples, one spot and one early morning, were routinely collected and used for acid-fast bacilli smear, culture examination, and TB detection by giant African pouched rats. Following NALC-NaOH decontamination, each sputum sample pellet was subjected to microscopy after Ziehl-Neelsen staining. Sputum smear were graded according to the concentration of the bacilli [18]. Each sample was inoculated on both liquid (BACTEC MGIT 960, Becton Dickinson, USA) and solid Löwenstein-Jensen (LJ) culture media. Positive cultures were confirmed by microscopy for acid fast bacilli and for the presence of *M*. *tuberculosis* by MPT64 antigen and/or molecular tests (Genotype MTBC, CM, or MTBDRplus, Hain Lifescience, Germany). Genotype CM or AS (Hain Lifescience, Germany) were used for detection of NTM. GenoType MTBDRplus or phenotypic drug-susceptibility testing (BACTEC MGIT 960 SIRE kit, Becton Dickinson, USA) was employed for resistance testing. All tests were performed blinded to clinical or radiological information by qualified laboratory technical personnel according to Good Clinical Laboratory Practice. Results from established diagnostic procedures were made available to support clinical management according to national and international guidelines.

If the volume of the specimen was appropriate (≥ 2ml), an aliquot of 1ml was collected from each unprocessed sputum samples in sterile cryovials before decontamination. The cryovials were stored within 4 hours at minus 20°C. The specimens were transferred under controlled temperature in one shipment to the APOPO laboratory in Morogoro, United Republic of Tanzania. After thawing, a sterile phosphate buffered saline was added and subsequent heat inactivation (90°C water bath for 30 min) was performed prior to detection by the giant African pouched rats.

### TB rats—training and detection sessions

Seven giant African pouched rats were used in succession for the detection of TB in the sputum samples. The animals had been trained prior to this study using operant conditioning to pause for at least 5 seconds over TB-positive samples (indicator response) and have passed an internal accreditation process under blind conditions. Training methods, standardisation of performance, quality control and experimental setup have been detailed before [5][7][19].

Prior to the detection sessions, training sessions with 196 sputum samples of known classification from the Ifakara Health Institute cohorts were carried out to allow adaptation to potential influence by factors specific to the site and the materials used (e.g. sputum collection container).

In the detection sessions, the personnel involved in the experiment at the APOPO laboratory were blind to the clinical, radiological, and mycobacteriological information related to the sputum samples. The samples (one or two per patient) were presented in detection sessions among samples that were being evaluated for routine second-line screening operations between 10^th^ July and 28^th^ August 2012.

### Statistical analysis and reference standard

In the main per-patient analysis, a TB-positive test result was defined as a positive indication by at least one rat of at least one sample of the patient. In this analysis, diagnostic test performance (sensitivity, specificity, predictive values and likelihood ratios) was calculated only in the groups with defined TB status (reference standard): group A (s+/c+ *M*.*tb*), group B (s-/c+ *M*.*tb*), and group F (controls). The performance has been analysed individually for each of the rats. Additionally, the performance of all 7 rats combined was analysed using the sum of the number of rats indicating a TB-positive test result (range 0 to 7) to determine the best threshold; e.g. a patient was considered test-positive if two or more rats indicated either of the samples of the patient as positive. Moreover, the diagnostic test performance was also assessed in a per-sample analysis. In this analysis the reference standard was defined as presence or absence of *M*.*tb* culture-positivity in the corresponding culture sample.

Receiver operating characteristic (ROC) curves and the areas under the curve (AUC) were calculated. Proportions were compared using logistic regression models and chi-square test. The statistical analysis was performed using Stata v13 (Stata Corp., College Station, TX, USA).

The presented diagnostic evaluation study followed guidelines of the TDR Diagnostics Evaluation Expert Panel (DEEP) and The Standards for Reporting of Diagnostic Accuracy (STARD) steering committee for assessing the test performance and operational features of diagnostics for infectious diseases in the respective target population [20][21].

### Ethical considerations

The study protocol and the consent procedure of the TB cohort and TB CHILD study were approved by the Institutional Review Board of the the Ifakara Health Institute and the Medical Research Coordinating Committee of Tanzania. Written informed consent was obtained from a literate patient. In case of illiteracy, informed oral consent was attested by an independent witness in accordance with Good Clinical Practice (GCP) guidelines [22]. In both cases the informed consent was documented on a paper-based, dated, signed and/or thumb-printed consent form. The study was conducted in accordance with the Helsinki Declaration [23] and GCP guidelines [22]. The Medical Research Coordinating Committee of Tanzania has granted ethics clearance for the use of African giant pouched rats as a potential tool for diagnosis of tuberculosis. APOPO has an approved Animal Welfare Assurance from the Office of Laboratory Animal Welfare (OLAW; Assurance Identification Number A5720-01).

## Results

A total of 480 individuals with symptoms suggestive of TB were enrolled; six children and adolescents of less than 15 years of age, one patient who was classified as having exclusively extrapulmonary TB and four patients without rat results were excluded from analysis. The 469 eligible study participants were assigned to the classification groups as displayed in Table 2.

**Table 2:** Patient characteristics and symptoms at recruitment.

	Missing data	All	Group A	Group B	Group C	Group D	Group F	Group G
			Culture-positive, smear-positive, *M*. *tuberculosis*	Smear-negative, culture-positive, *M*. *tuberculosis*	Culture-positive, non-tuberculous mycobacteria	Culture-negative pulmonary TB (strong clinical and radiological suspicion)	Controls, all smear- / culture-negative, sustained recovery up to 5th month	Loss to follow-up after recruitment or any other combination
**Characteristics**								
n (%)		469	80 (17.1)	29 (6.2)	84 (17.8)	9 (1.9)	128 (27.3)	139 (29.4)
Age Mean (SD)	0	41.6 (15.4)	36.8 (12.3)	42.3 (12.7)	43.2 (15.4)	44.9 (10.9)	42.3 (16.3)	42.3 (16.7)
Age Median (IQR)	0	38 (30–50)	35 (29–41)	39 (35–43)	42 (30–53.5)	45 (38–51)	39 (29.5–54.5)	39 (30–50)
Female sex—n (%)	1	231 (49.2)	25 (31.3)	16 (55.2)	51 (60.7)	5 (55.6)	64 (50.0)	70 (50.7)
Body mass index (kg/m^2^) Median (IQR)	2	19.7 (17.7–22)	18.6 (16.9–20)	18.9 (17.4–21.8)	19.8 (17.7–22.4)	20.2 (16.1–22.2)	20.6 (18.2–23.3)	19.4 (17.6–21.7)
HIV positive—n (%)	31	203 (46.4)	26 (34.7)	24 (82.8)	40 (51.3)	3 (37.5)	48 (40.7)	62 (47.7)
History of TB—n (%)	1	74 (15.8)	12 (15.0)	2 (6.9)	16 (19.1)	4 (44.4)	13 (10.2)	27 (19.6)
**Symptoms at recruitment**								
Cough ≥ 2 weeks—n (%)	1	442 (94.4)	74 (92.5)	26 (89.7)	80 (95.2)	9 (100)	124 (96.9)	129 (93.5)
Night sweats—n (%)	1	217 (46.4)	46 (57.5)	12 (41.4)	37 (44.1)	7 (77.8)	48 (37.5)	67 (48.6)
Haemoptysis—n (%)	9	52 (11.3)	8 (10.0)	1 (3.5)	11 (13.6)	1 (11.1)	14 (11.3)	17 (12.4)
Fever—n (%)	1	241 (51.5)	49 (61.3)	18 (62.1)	44 (52.4)	5 (55.6)	60 (46.9)	65 (47.1)
Weight loss—n (%)	4	229 (49.3)	50 (62.5)	19 (65.5)	35 (41.7)	3 (33.3)	56 (43.8)	66 (48.9)

About half of the participants were female (49%) and the overall HIV-prevalence was 46%. The proportion of HIV-infected participants was statistically significant higher in group B (s-/c+ *M*.*tb*) compared to all other groups (chi-square 22.21, p<0.001). Details on patient characteristics and symptoms at recruitment are shown in Table 2.

### *M*.*tb*: per patient analysis

The analysis of the combined diagnostic capability showed that sensitivity decreased with increasing threshold (indication by ≥1 to 7 rats) and, as an opposing trend, specificity increased. At the lowest and highest thresholds, sensitivities and specificities amounted to 71.6% (95% CI 62.1–79.8), 59.4 (95% CI 50.3–68.0) and 18.4% (95% CI 11.6–26.9), 97.7% (95% CI 93.3–99.5), respectively (Table 3).

**Table 3:** Diagnostic test performance of TB detection rats (per-patient analysis; all samples).

Culture-positive TB cases (Group A&B) versus Controls (Group F)	Threshold (Number of rats indicating TB)	Sensitivity	Specificity	Positive Predictive Value	Negative Predictive Value	Positive Likelihood Ratio	Negative Likelihood Ratio
N = 237		% (95% CI)	% (95% CI)	% (95% CI)	% (95% CI)	% (95% CI)	% (95% CI)
**Rat 1**		42.2 (32.8–52.0)	89.8 (83.3–94.5)	78.0 (65.3–87.7)	64.6 (57.1–71.6)	4.16 (2.37–7.28)	0.64 (0.54–0.76)
**Rat 2**		31.2 (22.7–40.8)	93.0 (87.1–96.7)	79.1 (64.0–90.0)	61.3 (54.1–68.2)	4.44 (2.23–8.83)	0.74 (0.65–0.85)
**Rat 3**		42.2 (32.8–52.0)	83.6 (76.0–89.5)	68.7 (56.2–79.4)	62.9 (55.2–70.2)	2.57 (1.64–4.03)	0.69 (0.58–0.83)
**Rat 4**		42.2 (32.8–52.0)	87.5 (80.5–92.7)	74.2 (61.5–84.5)	64.0 (56.4–71.1)	3.38 (2.03–5.61)	0.66 (0.56–0.79)
**Rat 5**		39.4 (30.2–49.3)	85.9 (78.7–91.4)	70.5 (57.4–81.5)	62.5 (54.9–69.7)	2.81 (1.72–4.57)	0.70 (0.60–0.83)
**Rat 6**		57.8 (48.0–67.2)	78.9 (70.8–85.6)	70.0 (59.4–79.2)	68.7 (60.5–76.1)	2.74 (1.89–3.97)	0.53 (0.42–0.68)
**Rat 7**		31.2 (22.7–40.8)	89.8 (83.3–94.5)	72.3 (57.4–84.4)	60.5 (53.2–67.5)	3.07 (1.71–5.52)	0.77 (0.67–0.88)
**Mean**		40.9 (37.5–44.3)	86.9 (85.2–88.7)	73.3 (71.7–74.8)	63.5 (62.0–64.5)	3.31 (3.03–3.59)	0.68 (0.65–0.71)
	≥1	71.6 (62.1–79.8)	59.4 (50.3–68.0)	60.0 (51.0–68.5)	71.0 (61.5–79.4)	1.76 (1.38–2.24)	0.48 (0.34–0.67)
	≥2	56.9 (47.0–66.3)	80.5 (72.5–86.9)	71.3 (60.6–80.5)	68.7 (60.6–76.0)	2.91 (1.98–4.29)	0.54 (0.42–0.68)
	≥3	47.7 (38.1–57.5)	88.3 (81.4–93.3)	77.6 (65.8–86.9)	66.5 (58.8–73.5)	4.07 (2.43–6.81)	0.59 (0.49–0.72)
**All rats combined**	≥4	36.7 (27.7–46.5)	92.2 (86.1–96.2)	80.0 (66.3–90.0)	63.1 (55.8–70.0)	4.70 (2.47–8.95)	0.69 (0.59–0.80)
	≥5	29.4 (21.0–38.8)	95.3 (90.1–98.3)	84.2 (68.7–94.0)	61.3 (54.2–68.1)	6.26 (2.72–14.42)	0.74 (0.65–0.84)
	≥6	25.7(17.8–34.9)	95.3 (90.1–98.3)	82.4 (65.5–93.2)	60.1 (53.0–66.9)	5.48 (2.36–12.74)	0.78 (0.69–0.88)
	7	18.4 (11.6–26.9)	97.7 (93.3–99.5)	87.0 (66.4–97.2)	58.4 (51.5–65.1)	7.83 (2.39–25.64)	0.84 (0.76–0.92)

CI = Confidence interval.

**Fig 1:**
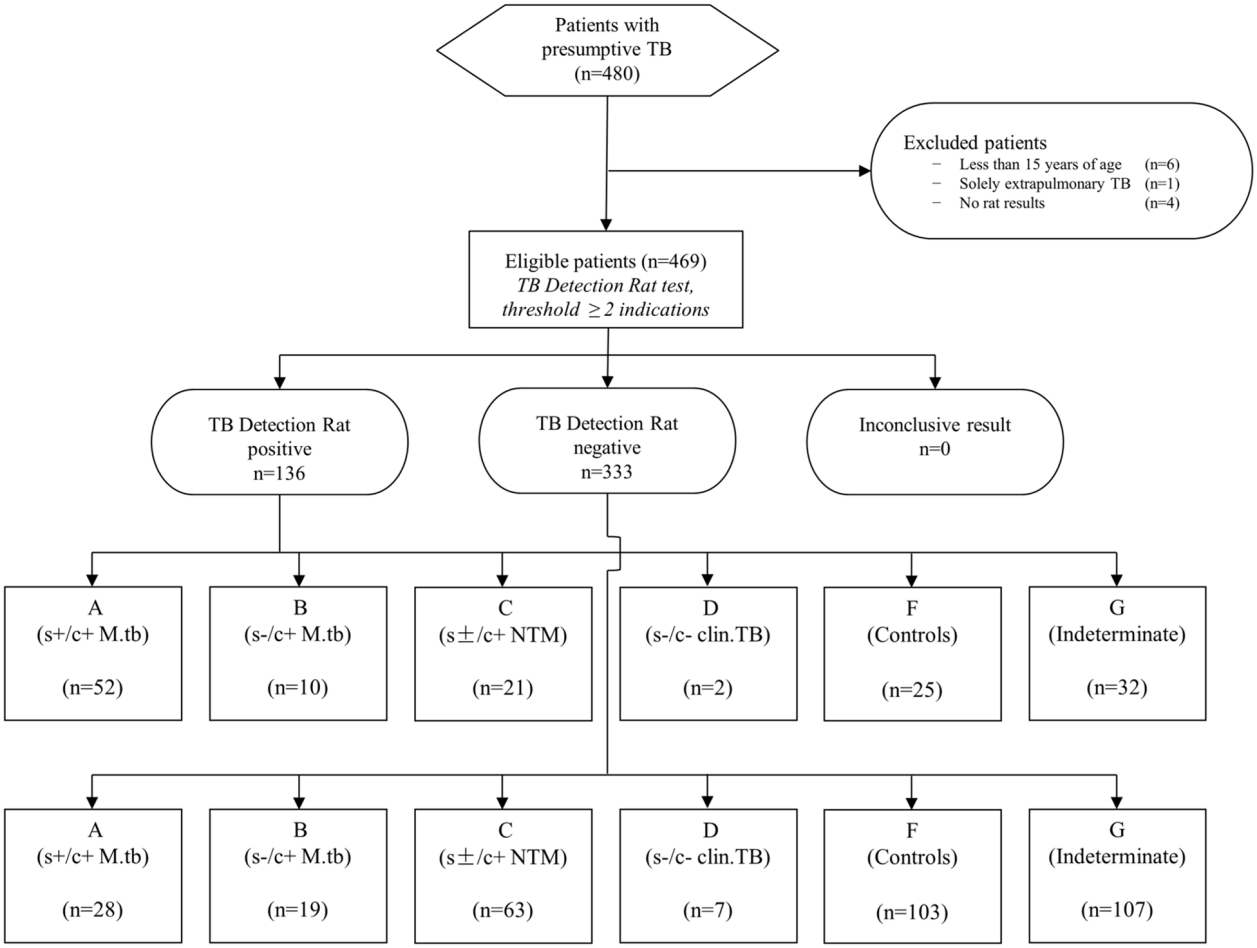
STARD flow chart for diagnostic threshold of ≥2 indications by rats.

**Fig 2:**
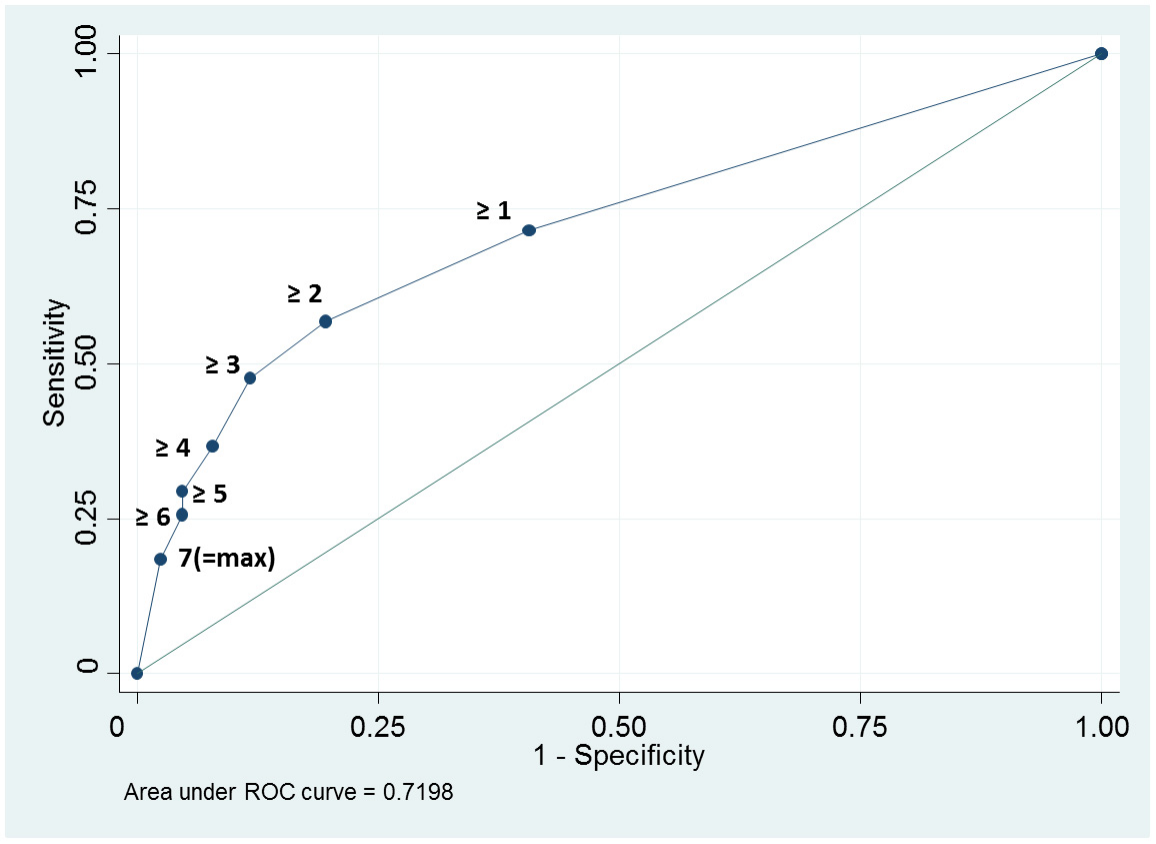
ROC analysis for the detection of *M*.*tb* culture-positive individuals for different indication thresholds (per-patient analysis; all samples). (Standard error 0.03, 95 CI% 0.66–0.78).

In the ROC calculation, the optimal diagnostic threshold (minimal square of distance between the upper left hand corner of ROC and any point on the ROC curve) could be defined as ≥2 indications by rats. Fig 1 shows the STARD flow diagram for this optimal threshold. The area under the ROC curve, which determines the inherent validity of the diagnostic approach, was 0.72 (95 CI% 0.66–0.78) for the detection of *M*.*tb* culture-positive individuals (Fig 2). For the optimal threshold, this translates to a sensitivity of 56.9% (95% CI 47.0–66.3), specificity of 80.5% (95% CI 72.5–86.9), positive (PPV) and negative predictive value (NPV) of 71.3% (95% CI 60.6–80.5) and 68.7% (95% CI 60.6–76.0), and an accuracy for TB diagnosis of 69.6%.

**Fig 3:**
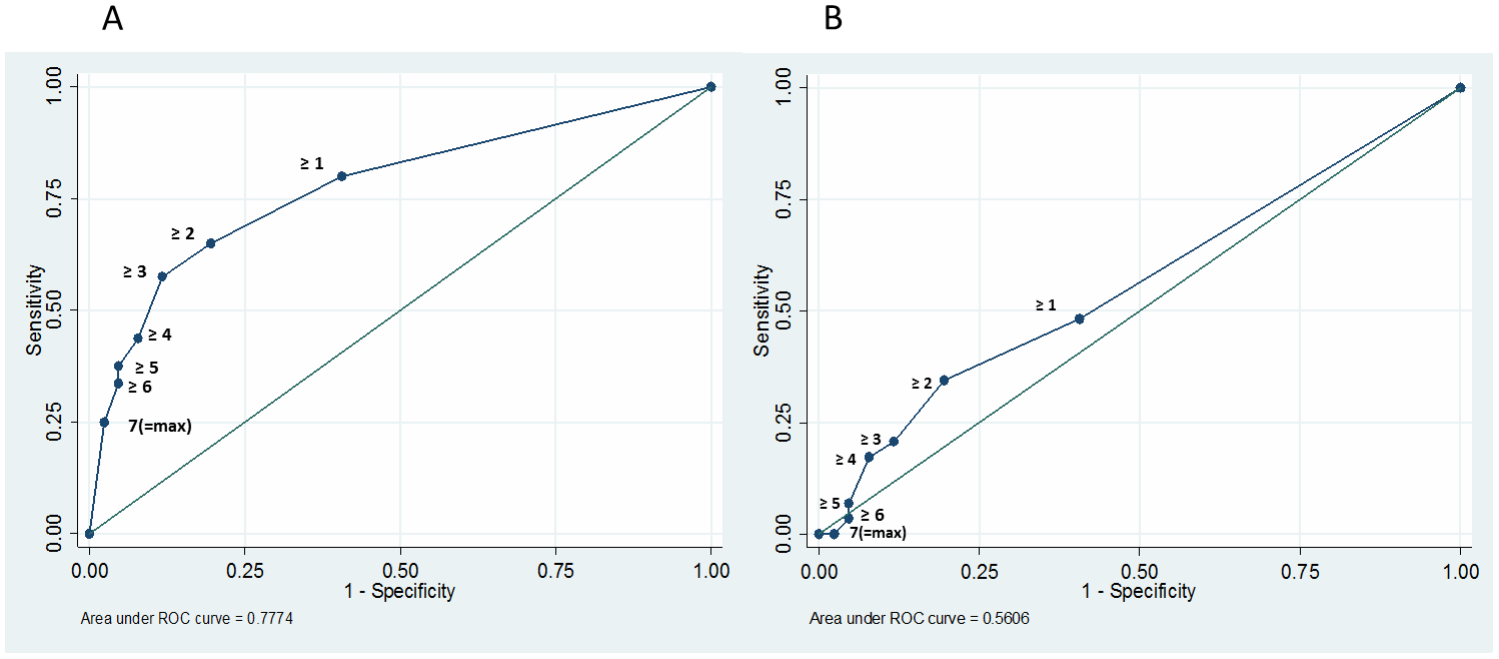
ROC analysis for the detection of smear-positive (A) versus smear negative (B) *M*.*tb* culture-positive individuals for indication thresholds (per-patient analysis; all samples). (A: Standard error 0.03, 95 CI% 0.71–0.84; B: Standard error 0.06, 95 CI% 0.45–0.67).

The area under the ROC curve differed statistically significant for detection of smear-positive (p = 0.016), *M*.*tb* culture-positive individuals and smear-negative, *M*.*tb* culture-positive individuals (0.78 vs. 0.56; Fig 3). For the optimal threshold, this translates to a sensitivities of 65.0% (95% CI 53.5–75.3) and 34.5% (95% CI 17.9–54.3) and specificities of 80.5% (95% CI 72.5–86.9) and 80.5% (95% CI 72.5–86.9) for the detection of smear-positive and smear-negative TB, respectively.

One sputum sample was collected from 122 (26%) patients and two samples were obtained from 347 (74%) patients. All available samples were tested by TB detection rats. The area under the ROC curve (graph not shown) did not differ if the rats tested only the first or both available samples (0.74 vs. 0.76).

There was no statistically significant difference in the performance of TB detection rats comparing the area under the ROC curves stratified by HIV status for group A (s+/c+ *M*.*tb*) and B (s-/c+ *M*.*tb*) and group F (control) as reference standards (chi-square 0.3466, p-value = 0.557).

Association analyses were performed under the assumption that ≥2 indications by rats represent the optimal threshold for TB detection. No statistically significant association was found between TB detection by rats and age, TB history, or having a cough, hemoptysis, sweating, or weight loss at recruitment. Having a fever at the time of recruitment was significantly associated with TB detection by rats (p = 0.003).

The diagnostic performance differed considerably between the individual rats, e.g. a statistically significant higher sensitivity was achieved by rat no.6 (57.8%; 95% CI 48.0%-67.2%) compared to rats no.2 and no.7 (31.2%; 95% CI 22.7%-40.8%). The specificity for six of the seven rats was above 80% (Table 3).

### *M*.*tb*: per sample analysis

In a per-sample analysis (n = 819), using presence or absence of *M*.*tb* growth in the culture of the same sample as reference standard, the indicators of test performance, in particular the sensitivity, were overall poor. Depending on the threshold, the sensitivity ranged from 45.8% (95% CI 40.3–51.4) to 5.9% (95% CI 3.6–9.0) and specificity from 73.8 (95% CI 69.7–77.7) to 99.0 (95% CI 97.6–99.7). The area under the ROC curve for the per-sample analysis was 0.61.

A per-sample sub-analysis in group A (s+/c+ *M*.*tb*; n = 134) showed a statistically significant association between grading of sputum smear and TB detection by rats with higher AFB results having more TB detection (chi-square 17.31, p = 0.002).

### NTM: sub-analysis

Group C (s ±/c+ NTM) encompassed patients with exclusively NTM in the sputum sample irrespective of a potential clinical relevance. In this group, 21 of 84 patients (25%) had a positive rat result (at least one sample with ≥2 indications). The per-sample analysis exhibited that 19.6% of the samples with concurrent identification of one or more NTM were rat-positive using the threshold of ≥2 indications. The proportion of rat-positive samples in the samples with the identified NTMs is displayed in Table 4.

**Table 4:** Rat-positive samples (two or more indications) in group C (s ±/c+ NTM) with concurrent molecular test result (per-sample analysis).

Molecular differentiation of non-tuberculous mycobacteria	≥2 rat indications
*M*. *abscessus/M*. *immunogenum*	0/1
*M*. *asiaticum*	3/10
*M*. *avium*	0/2
*M*. *celatum*	0/2
*M*. *fortuitum 1*	2/13
*M*. *fortuitum 2/M*. *mageritense*	1/12
*M*. *godii*	0/1
*M*. *gordonae*	1/2
*M*. *intracellulare*	4/15
*M*. *malmoense*	0/1
*M*. *malmoense/M*.*haemophilum/M*. *patustre*	0/2
*M*. *scrofulaceum*	1/7
*M*. *scrofulaceum and M*.*celatum*	0/1
*M*. *simiae*	2/5
*M*. *smegmatis*	0/2
*M*. *szulgai/M*.*intermedium*	1/1
High GC Gram positive bacterium	2/7
Mycobacterium species*	1/16

*Species could not be determined by Hain GenoType Mycobacterium CM/AS

## Discussion

Giant African pouched rats can detect M. tuberculosis in clinical sputum samples with a fair sensitivity (56.9%) and moderate specificity (80.5%) compared to culture as reference standard if an indicator response from two or more out of seven rats is treated as a positive diagnostic indicator.

This is the first evaluation of the diagnostic performance of TB detection rats which includes microbiological investigations with liquid and solid culture, subsequent differentiation of species, combined with a comprehensive clinical data set from each patient. Solid culture (LJ) has been used as reference standard in previous studies on TB detection rats either without [5] or with identification by subsequent multiplex real-time PCR [10][24]. However, detailed clinical data were not available in any of those studies. Dissimilarities in study design impede the direct comparison between the presented and former studies. In one study with culture and multiplex PCR using 10 instead of 7 rats [10], the per-patient sensitivity was higher (81.9% vs. 56.9%) and the specificity was lower (64.5% vs. 80.5%) at a diagnostic threshold of ≥2 indications. The difference in diagnostic accuracy at the defined threshold is most probably influenced by the different total number of rats used, resulting in a trade-off in sensitivity and a gain of specificity at the threshold of ≥2 in the presented compared to the previous study.

The presented data suggest that the performance of the TB detection rats depends on the bacterial load in sputum samples. TB was detected with a higher sensitivity in smear positive compared to smear negative, culture-positive TB patients. Moreover, the detection was significantly better in sputum samples with higher microscopy grade, a surrogate for high concentration of bacilli.

In general, the diagnostic performance was equally good if only one or two samples were used for detection.

Interestingly, the sensitivity and specificity for detecting TB did not differ significantly between HIV-infected and HIV-uninfected patients, although in general sputa of HIV-infected patients have a lower burden of TB bacilli [25][26]. Further HIV-related differences in symbiotic or pathogenic respiratory and oral microorganisms [27][28][29] with different bouquets of volatiles, seem to have no impact on diagnostic performance of the giant African pouched rats.

The seven rats did not perform with the same diagnostic accuracy: specificity and particularly sensitivity varied considerably between the individual rats. The findings contrast those of preceding studies [5][6] which report consistent performance characteristics for each rat. Tentatively, different levels of operant conditioning training, but also other influences, e.g. related to the patient group, might be accountable for the observed variability.

In previous studies, the rats detected TB in the same sputum containers which were earlier also used for collection at the microscopy centres. Yet, in the presented study the specimens have been transferred to cryovials before frozen, thawed and subsequently evaluated by the rats. It remains speculative, if the background odour of the container used for sputum collection has any potential effect on the diagnostic performance of the rats. According to unpublished data, the intermediate freezing of specimens does not seem to have an influence on the diagnostic performance of the TB detection rats [10].

The odour of *M*.*tb* does not consist of a single compound but rather a combination of volatiles which is characteristic for *Mt*.*b* (‘smellprint’) and does, with regard to many volatiles, not overlap with compounds of non-tuberculous mycobacteria or other pathogenic and apathogenic microorganisms of the respiratory tract [11][30][31]. In approximately 80% of the samples with NTM only, the rats did not indicate for TB in the presented clinical evaluation. The findings suggest that trained giant African pouched rats can discriminate *M*.*tb*-specific volatile compounds from non-tuberculous odours to a certain degree. This has been also similarly demonstrated in comprehensive experiments on cultured microbes and clinical sputa from microscopy centres [9][24].

Since one rat can screen 140 sputum samples in 40 minutes [5], large-volume second-line screening of sputum samples from microscopy centres in combination with a confirmatory test with high specificity could be a potential cost-effective application of detection rats technology. The high sample throughput and low cost make TB detection rats also a possible technology for active TB case finding in populations at high risk for TB, such as prison populations. However, the sensitivity would need to be further optimised, before TB detection rats can be used systematically for TB screening [32].

A limitation of the study is that the giant African pouched rats were primarily trained to perform an operant discrimination on high-intensity stimuli (high concentration of bacilli). This could explain the tendency towards superior detection in this kind of specimen. At the time of this publication, rats are now primarily trained sputum samples with low concentration of bacilli to improve performance with low concentration samples.

The objectives of this study were limited to the assessment of test accuracy in adult individuals with presumptive TB. Operational issues regarding a routine implementation of the technology on microscopy level and a potential scale up to different settings or countries with high burden of TB have not been addressed. In future, these research questions will become increasingly relevant, because until now breeding, training and diagnostic performance of TB detection rats has been only managed by APOPO, a non-governmental organisation using rats for TB detection in Tanzania and Mozambique.

In summary, giant African pouched rats have the potential for detection of tuberculosis in sputum samples. However, the diagnostic performance characteristics in this clinical evaluation were less favourable than those reported in previous diagnostic studies in less- or non-characterised patients [5][8][10][24].

Based on current evidence, rat technology as a standalone diagnostic test, does not fulfil the criteria for a rapid sputum-based test for detecting TB at the microscopy-centre level of the health-care system as it has been defined by the World Health Organization in 2014 at a stakeholder meeting on high-priority target product profiles [33][34].

Future efforts in research and development should be used to further improve the rat technology, to explore prospects for implementation and scale up and to determine its potential position in TB diagnostic or screening algorithms.
